# Pharmacists’ Experiences on Adverse Drug Reactions in Saudi Arabia: A Cross-Sectional Study

**DOI:** 10.3390/pharmacy13030087

**Published:** 2025-06-19

**Authors:** Dalal Salem Bakheit Aldossari, Naeema Taha Alshalaan, Khuloud Salem Alshammari, Fatima Ahmed Lubbad, Mudhi Ratyan Alanazi, Neamah Ahmed Lubbad, Nada Suliman Fahad Alessa, Sheraz Ali

**Affiliations:** 1 Pharmaceutical Care Services, King Saud Medical City, Riyadh 12746, Saudi Arabia; d.dawsari@ksmc.med.sa (D.S.B.A.); omfahad2@yahoo.com (N.T.A.); kalshammery@ksmc.med.sa (K.S.A.); f.lubbad@ksmc.med.sa (F.A.L.); modi@ksmc.med.sa (M.R.A.); dr.neamah@ksmc.med.sa (N.A.L.); nalessa@ksmc.med.sa (N.S.F.A.); 2 Institute for Evidence-Based Healthcare, Faculty of Health Sciences & Medicine, Bond University, Gold Coast 4226, Australia

**Keywords:** adverse drug reactions (ADR), pharmacovigilance, pharmacists’ experiences, Saudi Arabia, hospital pharmacists, community pharmacists, drug safety

## Abstract

Background and objectives: As part of Vision 2030, Saudi Arabia aims to strengthen its healthcare system by enhancing efficiency, reducing medical errors, and ensuring drug safety. Evidence on pharmacists’ experiences with adverse drug reactions (ADRs) in daily practice remains limited. Gaining insight into their perspectives is essential for improving patient safety and optimizing pharmaceutical care. Therefore, we aimed to assess pharmacists’ ability to identify ADRs in daily practice and the subsequent actions taken upon identification. Methods: Between July and August 2024, an email-based invitation was sent to randomly selected registered community and hospital pharmacists in Saudi Arabia to participate in the study, which employed a piloted questionnaire. Results: The study involved 305 pharmacists, including 169 hospital/clinical pharmacists (HCPs, 55.4%) and 136 community pharmacists (CPs, 44.6%). A majority (*n* = 251, 82.3%) indicated direct patient encounters, while 67.2% (*n* = 205) reported observing suspected ADRs in the preceding 12 months. Most respondents filed ADR reports to the Saudi Food and Drug Administration/National Pharmacovigilance Centre (HCP = 103, CP = 60) and hospital drug information centers (HCP = 89, CP = 64), with online forms being the favored mode (HCP = 122, CP = 96). Awareness of ADR reporting procedures was reported by 128 HCPs and 80 CPs. Conclusions: More than two-thirds of participants reported having participated in ADR reporting, with greater adherence observed in hospital settings. Pharmacists predominantly depend on the Saudi Food and Drug Administration/National Pharmacovigilance Centre and hospital drug information centers for reporting, with a preference for online submission methods. Targeted educational interventions addressing gaps in knowledge, reporting procedures, and form complexity could improve ADR reporting practices. These findings support the need for structured training and policy measures to strengthen pharmacovigilance system.

## 1. Introduction

The World Health Organisation (WHO) defines adverse drug reaction (ADR) as “a response to a drug that is noxious and unintended, and that occurs at doses that are typically used in patients for the prophylaxis, diagnosis, or therapy of disease, or for the modification of physiological function” [1]. ADR is a significant public health concern that is linked to high morbidity and mortality rates, resulting in increased healthcare expenditure, unnecessary readmissions, and the prolongation of hospitalization [2,3]. Therefore, it is imperative to implement post-marketing surveillance to oversee ADRs associated with novel pharmaceuticals [3].

The reporting of ADRs that occur spontaneously is the primary method of monitoring newly marketed medications. Following the thalidomide incident, the WHO implemented the International Programme for Adverse Drug Reaction Monitoring to oversee global drug safety [4]. WHO, in collaboration with the European Medicines Agency and the United States Food and Drug Administration (FDA), has advanced the regulatory practice that safeguards the global community [4]. The Netherlands, United Kingdom, and Denmark were the first countries to implement spontaneous reporting systems in the 1960s [5]. Numerous nations subsequently adopted this approach. Saudi Arabia’s National Pharmacovigilance Centre is responsible for the submission of all suspected ADRs in Saudi Arabia [6]. These cases are subsequently submitted to the Uppsala Monitoring Centre in Sweden for inclusion in the WHO database [6]. Despite the existence of an ADR reporting system for decades, the issue of underreporting ADRs persists [6,7].

In comparison to other healthcare personnel, pharmacists are highly regarded for their primary responsibilities of reporting adverse drug reactions (ADRs) and implementing pharmacovigilance (PV) principles in their daily clinical practice [8,9]. Most pharmacists are knowledgeable of the existence of a reporting system; however, only a small number of pharmacists have filed a report [10]. Evidence indicates that pharmacists lack knowledge regarding the process of reporting an ADR or confidence in which ADRs to report [5]. This underscores the necessity of pharmacists receiving a comprehensive education in pharmacovigilance and spontaneous ADR reporting systems [9].

The under-reporting of ADRs is a major problem across heath care professionals [11]. For example, a systematic review of studies conducted in the European Union showed a significant and widespread trend of healthcare professionals (HCPs) under-reporting ADRs, with a median rate of under-reporting of 94% [12]. Under-reporting is directly linked to the knowledge, attitude, and practice of healthcare professionals and the availability of ADR reporting systems [12].

Given the legal obligation for pharmaceutical companies and healthcare professionals to report suspected adverse drug reactions (ADRs) in Saudi Arabia, the ADR reporting behaviors of pharmacists and other healthcare professionals have been the primary focus of previous studies in Saudi Arabia [13,14,15]. Nevertheless, there is a significant gap in the evidence that pertains to the experiences of pharmacists with ADRs in their daily clinical practice. Therefore, our primary aim was to determine whether pharmacists could identify and report ADRs during their daily routine. Secondarily, we aimed to assess the perceived challenges and facilitators of ADR reporting. The findings of this research can serve as a benchmark for stakeholders to implement a strategy that enhances pharmacists’ knowledge, attitude, and practices in the identification and reporting of ADRs.

## 2. Methods

### 2.1. Study Design and Setting

This study was questionnaire-based and conducted between July 2024 and August 2024. Registered pharmacists employed at hospitals or community pharmacies in Saudi Arabia comprised the population. Data collection was conducted utilizing Google Forms.

### 2.2. Study Tool

The survey was adopted from a previous study with slight revisions to improve relevance and comprehensiveness [5]. A pre-test was undertaken on 10 pharmacists. Based on piloting feedback, we re-evaluated the questionnaire to make sure it was easy to read and complete. The final review was made by a pharmacist for face validity. Based on the pre-test feedback, we determined the completion of the survey to take ten minutes. The questionnaire comprised two sections and a total of 16 items. There were three types of questions: dichotomous (Yes/No), multiple-choice, and multiple-response (check-all-that-apply), with open-ended options provided when appropriate. Attitude assessment items covered topics such as preferred reporting methods, perceived goals of spontaneous reporting, and factors that encourage or discourage ADR reporting. Section 1 of the survey asked participants to identify themselves by occupation, number of years in the field, and whether they had direct patient contact. The second section inquired about the respondents’ encounters with ADRs. Among the questions asked were the frequency of suspected ADR cases seen in the previous six months and whether they had seen any in the last twelve months. The National Pharmacovigilance Centre and the related literature informed the predetermined list that respondents were instructed to use when questioned about the types of ADR symptoms encountered. There was an open-ended option for participants to report any symptoms that were not included, and multiple responses were allowed. Similarly, for each ADR case that participants observed, we gave them a list of medications that are often implicated and asked them to check all that applied. We included an open-response option to allow participants to add any medicines that were not already listed. Pharmacists’ responses to the reported ADRs were also investigated in the survey. The participants had the ability to submit additional responses not included in the list, and they had the option to choose from a range of predefined actions based on the current literature and standards. Also, using checklists, we checked participant’s knowledge and attitudes toward ADR reporting. Respondents’ preferred means of reporting, their level of familiarity with reporting processes, their perceptions of obstacles and facilitators, and their views on which ADRs should be reported were all evaluated by these questions. Every question was constructed to be compatible with national pharmacovigilance standards and, when appropriate, to accommodate multiple choice answers.

Accordingly, they were provided with a list of adverse drug reaction (ADR) types and asked to select one or more that they believed should be reported. Likewise, a list of potential purposes for monitoring and reporting suspected ADRs was given, allowing them to choose one or more based on their knowledge and perceptions

### 2.3. Recruitment

The researchers disseminated the survey link to individuals in their personal network, including friends and coworkers through social media platforms such as Facebook, LinkedIn, Twitter, and WhatsApp. These participants were requested to distribute this survey to colleagues through social media platforms. Professional groups were given priority. A reminder email was sent after one week.

### 2.4. Ethical Considerations

This research was initiated after the ethics approval (Approval number: H1RI-26-Jun24-04) from the Institutional Review Board, King Saud Medical City, Saudi Arabia. The completion of the survey was considered as implied consent.

### 2.5. Sample Size and Data Analysis

A recent study reported that the total number of pharmacists in Saudi Arabia is 30,840 [16]. A sample size of 380 was estimated using the Raosoft calculator [17], taking into consideration the population size, a 95% confidence level, and a 5% margin of error. All data was subjected to descriptive statistics. To guarantee that the data was entered accurately and comprehensively, the frequencies of variables were calculated and verified for values that exceeded the permissible range. The pharmacist groups’ experiences with ADRs were compared using the Pearson chi-square test. All statistical analyses were conducted using IBM SPSS Statistics version 27. The level of significance was considered at *p* < 0.05.

## 3. Results

A total of 305 professionals were recruited for this study. Most of the study participants, 169, were employed as hospital or clinical pharmacists, while 136 others were community pharmacists. Only 4.9% of respondents gained more than 30 years of professional experience. In total, 39 percent of respondents had sixteen to thirty years of experience, 33 percent had six to fifteen years, and 36.4% had less than five years. When pharmacists were asked about their direct interactions with patients, the majority of the respondents (82.3%) reported that they had direct contact with the patients, while only 15.7% reported that they had no such contact. Within the previous year, two-thirds of the respondents reported that they had observed ADR suspects. Participants were asked to estimate their frequency of encountering patients with suspected ADRs in clinical practice. The reported frequency of encountering suspected ADR cases varied among participants: 41% experienced fewer than one case per-month, while 20.3%, 15.1%, and 20.3% reported encountering cases at least monthly, weekly, and daily, respectively (Table 1).

The current findings revealed a diverse range of suspected adverse drug reactions (ADRs) identified by pharmacists. Dermatological reactions were the most frequently reported, with rash (48.5%; *p* = 0.011) and itchiness (42.0%; *p* = 0.341) being the leading complaints. These were followed by respiratory-related ADRs, where dry cough (38.0%; *p* = 0.251) and cough (28.5%; *p* = 0.758) were the most commonly noted symptoms.

A Chi-square test indicated that statistically significant differences were observed in several specific ADRs between hospital or clinical pharmacists (HCPs) and community pharmacists (CPs). Palpitations (24.6%; *p* = 0.024), hyperkalemia (6.6%; *p* = 0.022), and hyperglycemia (6.2%; *p* = 0.033) were reported more frequently by HCPs, indicating potential differences in clinical exposure or awareness levels between the two groups (Table 2).

Regarding the actions taken by pharmacists in response to suspected adverse drug reactions (ADRs), the most commonly reported formal action was submitting a report to the Saudi Food and Drug Authority (SFDA)/National Pharmacovigilance Centre, cited by 53.8% of participants, with no statistically significant difference between hospital/clinical pharmacists (HCPs) and community pharmacists (CPs) (*p* = 0.100). Additionally, 50.2% of pharmacists reported ADRs to hospital drug information centers (*p* = 0.331), and 40.3% informed another hospital pharmacist (*p* = 0.971) (Table 3).

Regarding participants’ perspectives on sources of adverse drug reaction (ADR) reporting forms, a majority of hospital/clinical pharmacists (HCPs) (*n* = 123, 72.8%) and community pharmacists (CPs) (*n* = 78, 57.4%) reported obtaining the forms from the Saudi Food and Drug Authority (SFDA) website, with a statistically significant difference between the groups (*p* = 0.005). Additional reported sources included the hospital’s drug information center (HCP: 103 [60.9%], CP: 80 [58.8%]; *p* = 0.707), national or local health departments (HCP: 36 [21.3%], CP: 30 [22.1%]; *p* = 0.873), drug information texts (HCP: 24 [14.2%], CP: 16 [11.8%]; *p* = 0.531), and other miscellaneous sources (HCP: 13 [7.7%], CP: 15 [11.0%]; *p* = 0.316).

When asked about their preferred method for submitting ADR reports to the SFDA/National Pharmacovigilance Centre (NPC), both HCPs (*n* = 122, 72.2%) and CPs (*n* = 96, 70.6%) most frequently favored online form submission (*p* = 0.758). This was followed by the Saudi Vigilance mobile application (HCP: 83 [49.1%], CP: 56 [41.2%]; *p* = 0.167), phone reporting (HCP: 33 [19.5%], CP: 24 [17.6%]; *p* = 0.676), and email submissions (HCP: 41 [24.3%], CP: 27 [19.9%]; *p* = 0.358). Less commonly used methods included reporting exclusively to the hospital drug information center (HCP: 48 [28.4%], CP: 43 [31.6%]; *p* = 0.542), faxing forms (HCP: 17 [10.1%], CP: 19 [14.0%]; *p* = 0.293), and mail/postal submissions (HCP: 39 [23.1%], CP: 31 [22.8%]; *p* = 0.953) (Table 4).

Participants were also asked about their motivating and discouraging factors influencing them to report suspected adverse drug reactions (ADRs) to the Saudi Food and Drug Authority/National Pharmacovigilance Centre (SFDA/NPC). The severity of the clinical reaction was the most influential motivating factor, reported by 132 HCPs and 85 CPs, and was the only factor to show a statistically significant difference (*p* = 0.003). Other motivators included the explicit request of a pharmaceutical company (HCP = 78, CP = 58; *p* = 0.540), reaction not widely known (HCP = 78, CP = 62; *p* = 0.922), specific typology of the reaction (HCP = 32, CP = 36; *p* = 0.116), involvement of a newly licensed drug (HCP = 47, CP = 38; *p* = 0.980), and obvious causal relationship with drug administration (HCP = 33, CP = 29; *p* = 0.698), none of which were statistically significant.

Conversely, several deterrents to ADR reporting were identified. The most prominent included uncertainty regarding the type of reactions to be reported (HCP = 81, CP = 53; *p* = 0.117), uncertainty of a causal relationship with the drug administration (HCP = 52, CP = 47; *p* = 0.482), and low severity of reactions (HCP = 99, CP = 65; *p* = 0.060). Other barriers were lack of information from affected patients (HCP = 57, CP = 42; *p* = 0.598), lack of knowledge regarding reporting regulations and procedures (HCP = 44, CP = 20; *p* = 0.016), difficulty obtaining reporting forms (HCP = 36, CP = 32; *p* = 0.642), and complexity of the reporting form (HCP = 33, CP = 22; *p* = 0.449). Despite these factors being frequently reported, none except lack of knowledge of reporting regulations showed statistical significance (Table 5).

The study identified several types of adverse drug reactions (ADRs) perceived as reportable by pharmacists. Suspected reactions were considered reportable by 60.9% of hospital/clinical pharmacists (HCPs) and 55.1% of community pharmacists (CPs) (*p* = 0.307). Certain (ascertained) reactions were reportable to 58.0% of HCPs and 50.7% of CPs (*p* = 0.206), while severe reactions were deemed reportable by 52.1% of HCPs and 49.3% of CPs (*p* = 0.626). Mild reactions were less frequently regarded as reportable (32.5% HCPs vs. 25.0% CPs; *p* = 0.150). Reactions to drugs with long-term use were reportable to 26.6% of HCPs and 19.1% of CPs (*p* = 0.123). Reportability of reactions to new drugs was noted by 36.7% of HCPs and 30.1% of CPs (*p* = 0.230). Known reactions were less often reported (16.6% HCPs vs. 14.7% CPs; *p* = 0.657), whereas unexpected or unusual reactions were considered reportable by 39.6% of HCPs and 32.4% of CPs (*p* = 0.188). Other perceived reportable ADR types included drug interactions (24.9% HCPs, 23.5% CPs; *p* = 0.789), teratogenicity phenomena (31.9% HCPs, 25.0% CPs; *p* = 0.183), reactions to vaccination (37.9% HCPs, 26.5% CPs; *p* = 0.035), and lack of drug efficacy due to resistant strains (36.1% HCPs, 22.8% CPs; *p* = 0.012). Regarding the aims of spontaneous ADR reporting, measuring the incidence of ADRs was identified as the main objective by 76.3% of HCPs and 63.2% of CPs (*p* = 0.013). Other perceived aims included identifying drug prescription indications (60.4% HCPs, 55.9% CPs; *p* = 0.431), identifying predisposing factors for ADRs (55.0% HCPs, 47.8% CPs; *p* = 0.209), identifying uncommon ADRs such as allergic or idiosyncratic reactions (43.8% HCPs, 41.9% CPs; *p* = 0.742), identifying previously unknown ADRs (23.7% HCPs, 30.1% CPs; *p* = 0.203), assessing drug safety (38.5% HCPs, 36.0% CPs; *p* = 0.663), and maintaining ADR databases (44.9% HCPs, 38.2% CPs; *p* = 0.236) (Table 6).

## 4. Discussion

The current study aimed to explore the experiences of pharmacists working in both community and hospital settings with ADR reporting, as well as the barriers and facilitators influencing the reporting process in the Kingdom of Saudi Arabia. Over the course of a year, 70.5% of participants observed suspected ADRs, and of those, 53.4% reported the ADRs to the SFDA. Participants rated the high severity of the drug reaction as the most important factor encouraging them to report the ADR, followed by situations where the reaction is not widely known and the explicit request from a pharmaceutical company, in that order.

The observed increase in ADR reports compared to previous studies may be attributed to differences in the study settings. An analysis of the reported results shows that hospital pharmacists had higher ADR reporting rates compared to community pharmacists, although this difference was not statistically significant. The dominance of hospital pharmacists in pharmacovigilance activities has also been documented in a study conducted in Spain, where hospital pharmacists reported suspected ADRs far more frequently than their community pharmacist counterparts [18]. Earlier studies primarily focused on community pharmacies, where both clients and pharmacists are typically less engaged in ADR reporting [19,20]. In contrast, hospital pharmacies handle more severe ADR cases, which account for a significant proportion of hospital admissions [21]. Moreover, previous research has highlighted a lack of awareness among community pharmacists about the ADR reporting system in the country [22]. However, the current report aligns with a study from the UAE among hospital pharmacists, where 53.2% of participants reported suspected ADRs to the authority responsible [23]. Overall, both the current study and previous research highlight that the under-reporting of ADRs by pharmacists remains common in Saudi Arabia, emphasizing the need for continued efforts to enhance ADR reporting activities by all stakeholders.

The current study identified dermatological reactions—such as rash and itchiness—and respiratory symptoms—such as cough—as the most commonly suspected adverse drug reactions (ADRs) detected by pharmacists. This finding aligns with the results reported by Karuppannan et al. in Malaysia [5], where rash, itchiness, and dry cough were among the top ADRs reported by pharmacists. In addition to similarities in research design, the high visibility and recognizability of dermatological and respiratory symptoms may explain the consistency in these findings.

The current study also found that pharmacists’ interest in reporting increased when the suspected ADR is severe, the reaction is unknown, there is an explicit request from a pharmaceutical company, etc. Importantly, a relatively small proportion of participants (85 out of 305) identified newly introduced medicines as a motivating factor for reporting suspected adverse drug reactions (ADRs). However, the World Health Organization (WHO) strongly recommends establishing a strong and robust pharmacovigilance system for newly marketed medicines. Such systems are essential for evaluating the safety and benefit-risk profile of medicines and for supporting regulatory decision-making. This suggests a potential gap in the educational training of pharmacy professionals regarding the importance of pharmacovigilance, particularly for newly introduced medicines. Conversely, participants were less motivated to report when the suspected ADR is less severe, there is uncertainty about the type of reaction, or the causal relationship with the medication is unclear, etc. Similar findings were reported in the studies reviewed [24,25,26,27]. Pharmacists are more likely to report severe reactions due to the associated health risks and ethical and legal responsibilities. Additionally, the awareness of consequences gained during their academic career, along with continuous training on severe ADRs, serves as further motivation for pharmacists to report.

The current study explored participants’ viewpoints on which types of ADRs should be reported. Suspected reactions, confirmed reactions, and severe reactions were the most identified categories, along with other types. The current findings align with WHO recommendations, and national SFSA guideline which advocate reporting all clinically relevant suspected reactions. This includes suspected reactions to new drugs, increased frequency of known reactions, and unusual suspected ADRs associated with well-established medications [28,29]. Participants’ responses regarding the aims of ADR reporting emphasized identifying factors that expose patients to ADRs, detecting uncommon ADRs, and maintaining an ADR database as the most cited objectives. This aligns with guideline recommendations, which generally emphasize that ADR reporting helps assess drug safety in real-world conditions [29].

### Study Limitations

This study has several limitations. The cross-sectional design limits causal inference, and the self-reported questionnaire may not fully capture the complexity of pharmacists’ ADR reporting behavior, with potential recall and social desirability biases. Non-random sampling through networks and social media may have introduced selection bias, and respondent eligibility was not verified. Uneven group sizes and possible non-response bias may affect group comparisons. The absence of qualitative data and follow-up limits understanding of underlying motivations and actual reporting outcomes. In addition, the study involved a relatively small sample size, with a response rate of 80.3%, which may have affected the statistical power and the precision of the estimated results.

## 5. Conclusions and Recommendations

More than two-thirds of participants reported having participated in ADR reporting, with greater adherence observed in hospital settings. Pharmacists predominantly depend on the SFDA/NPC and hospital drug information centers for reporting, with a preference for online submission methods. These findings indicate the necessity to support and expand digital reporting platforms to improve ADR reporting efforts.

Integration of ADR reporting into pharmacy education, continuous professional development programs, or provision of mandatory workspace protocol would likely improve the knowledge gap and involvement in ADR reporting practice.

## Figures and Tables

**Table 1:** Demographic data of participated pharmacists (*N* = 305).

Demographic Variables	Frequency (*N*)	Percentage (%)
Profession		
Hospital/clinical pharmacist	169	55.4
Community pharmacist	136	44.6
Professional experience		
<5 years	111	36.4
6–15 years	102	33.4
16–30 years	76	24.9
More than 30 years	15	4.9
Missing	1	.3
Direct contact with patients		
Yes	251	82.3
No	48	15.7
Missing	6	2.0
Observed a suspect ADR last 12 months		
Yes	215	70.5
No	87	28.5
Missing	3	1.0
Observed a suspect ADR last 6 months		
At least 1 case per day	62	20.3
At least 1 case per week	46	15.1
At least 1 case per month	62	20.3
Less than 1 case per month	125	41.0
Missing	10	3.3

**Table 2:** Observed ADRs and implicated suspected medicines by study participants (*N* = 305).

System Organ Class	Observed ADR	HCP (*n* = 169)	CP (*n* = 136)	Toal Pharmacists (*N*)	Percentage (%)	*p*-Value
Respiratory	Cough	47	40	87	28.5%	0.758
	Dry cough	70	46	116	38.0%	0.251
Cardiovascular	Palpitation	50	25	75	24.6%	0.024
	Hyperkalemia	16	4	20	6.6%	0.022
	Bleeding	21	11	32	10.5%	0.219
	Edema	32	30	62	20.3%	0.500
	Thrombocytopenia	16	6	22	7.2%	0.090
Dermatological	Rash	93	55	148	48.5%	0.011
	Itchiness	75	53	128	42.0%	0.341
	Erythema	9	7	16	5.2%	0.945
	Steven Johnson Syndrome	8	4	12	3.9%	0.423
	Anaphylaxis reaction	24	14	38	12.5%	0.304
Gastrointestinal	Nausea	32	25	57	18.7%	0.902
	Vomiting	27	15	42	13.8%	0.213
	Diarrhea	25	15	40	13.1%	0.333
	Heartburn	20	12	32	10.5%	0.394
	Gastritis	17	17	34	11.1%	0.501
	Constipation	16	11	27	8.9%	0.673
	Flatulence	7	8	15	4.9%	0.485
Hepatic	Jaundice	8	3	11	3.6%	0.239
	Acute hepatitis	9	2	11	3.6%	0.073
Renal	Renal failure	9	5	14	4.6%	0.494
Neurological	Dizziness	38	40	78	25.6%	0.269
	Headache	54	36	90	29.5%	0.297
Musculoskeletal	Myalgia	17	13	30	9.8%	0.884
Endocrine/Metabolic	Hyperglycemia	15	4	19	6.2%	0.033
Drug Class	Suspected medicines *			Pharmacists (*N*)	Percentage (%)	
CCBs	Amlodipine	53	42	163	53.4%	0.929
	Nifedipine	44	24			0.080
ACE Inhibitors	Captopril	31	21	148	48.5%	0.503
	Perindopril	56	40			0.486
Antiplatelets	Aspirin	56	39	99	32.5%	0.403
	Ticlopidine	3	1			0.428
Statins	Atorvastatin	54	33	95	31.1%	0.139
	Lovastatin	5	3			0.683
Anticonvulsants	Phenytoin	6	4	88	28.9%	0.767
	Carbamazepine	51	27			0.040
NSAIDs	Diclofenac	37	34	78	25.6%	0.523
	Mefenamic acid	4	3			0.926
Penicillins	Amoxicillin	18	12	71	23.3%	0.594
	Cloxacillin	7	9			0.335
	Penicillin	15	10			0.630
Antidiabetics	Metformin	44	25	69	22.6%	0.112
Anticoagulants	Heparin	22	12	34	11.1%	0.247
Cephalosporins	Cefuroxime	17	1	33	10.8%	<0.001
	Ceftriaxone	7	8			0.485
Corticosteroids	Prednisolone	9	2	24	7.9%	0.073
	Dexamethasone	10	3			0.111
Analgesics/Antipyretics	Paracetamol	10	12	22	7.2%	0.329
Biologics	Infliximab	5	2	20	6.6%	0.388
	Adalimumab	8	5			0.650
Sulfonamides	Co-trimoxazole	10	8	18	5.9%	0.990
Diuretics	Chlorothiazide	9	9	18	5.9%	0.634
Retinoids	Isotretinoin	11	7	18	5.9%	0.616
Macrolides	Erythromycin	9	8	17	5.6%	0.833
Xanthine Oxidase Inhibitor	Allopurinol	10	5	15	4.9%	0.368
Beta-blockers	Atenolol	9	4	13	4.3%	0.306
Bisphosphonates	Alendronate	10	3	13	4.3%	0.111
TB Medications	Rifampicin	8	1	9	3.0%	0.040
Opioids	Morphine	5	2	7	2.3%	0.388
Immunomodulators	Fingolimod	3	1	4	1.3%	0.428

* Note: participants were made to select one or more medicines.

**Table 3:** Actions taken after identification of ADR, reporting status, and accessibility of reporting form by study participants (*N* = 305).

Category	Specific Action or Response		HCP (*n* = 169)	CP (*n* = 136)	Total Pharmacists (*N*)	Percentage (%)	*p*-Value
Actions Taken	Reported to Saudi FDA/National Pharmacovigilance Centre		98	66	164	53.8%	0.100
	Reported to hospital drug information center		89	64	153	50.2%	0.331
	Informed pharmacist at hospital drug information center		68	55	123	40.3%	0.971
	Informed physician or pharmaceutical company		48	43	91	29.8%	0.542
	Performed further evaluation (e.g., medication history review)		34	26	60	19.7%	0.827
	Noted in patient’s chart/record		41	31	72	23.6%	0.764
	Suggested patient inform their doctor		45	33	78	25.6%	0.638
	Suggested patient try a different medicine		22	27	49	16.1%	0.106
	Suggested patient stop the medicine		28	31	59	19.3%	0.171
	Suggested medicine to relieve the reaction		24	22	46	15.1%	0.632
	Explained to the patient it may be a reaction to their medicine		49	42	91	29.8%	0.720
	Took no action		14	4	18	5.9%	0.049
	Other		6	7	13	4.3%	0.493
ADR Reporting Status	Reported a suspected ADR to National Pharmacovigilance Centre	Yes	103	60	163	530.4%	0.005
		No	61	74	135	440.3%	
		Missing response	5	2	7	20.3%	
Access to Reporting Form	Has access to the official ADR reporting form	Yes	128	80	208	680.2%	0.007
		No	40	54	94	300.8%	
		Missing response	1	2	3	10.0%	

**Table 4:** Sources of ADR reporting form and preferred method to submit ADR report by study pharmacists (*N* = 305).

Item	HCP (*n* = 169)	CP (*n* = 136)	Total	Percentage (%)	*p*-Value
Sources for ADR reporting form					
From the Saudi Food and Drug Administration webpage	123	78	201	65.9%	0.005
From the hospital’s drug information center	103	80	183	60.0%	0.707
From national or local health department	36	30	66	21.6%	0.873
From the drug information book	24	16	40	13.1%	0.531
Other	13	15	28	9.2%	0.316
Preferred method to submit ADR report					
Filling in an online form	122	96	218	71.5%	0.758
Submit via Saudi Vigilance mobile application	83	56	139	45.6%	0.167
Reporting by phone	33	24	57	18.7%	0.676
Reporting by email at NPC.Drug@sfda.gov.sa	41	27	68	22.3%	0.358
Filling out a form and faxing it	17	19	36	11.8%	0.293
Mailing/posting an ADR report	39	31	70	23.0%	0.953
Reporting to the hospital’s drug information center only	48	43	91	29.8%	0.542

**Table 5:** Comparison of responses between hospital/clinical and community pharmacists factors encouraging and discouraging the reporting of ADRs.

Item	HCP (*n* = 169)	CP (*n* = 136)	Frequency	Percentage (%)	*p*-Value
Encouraging Factors					
The high degree of severity of a clinical reaction	132	85	217	71.1%	0.003
The explicit request of a pharmaceutical company	78	58	136	44.6%	0.540
The reaction is not widely known	78	62	140	45.9%	0.922
The specific typology of the reaction (unusual/unexpected)	32	36	68	22.3%	0.116
The involvement of a newly licensed drug	47	38	85	27.9%	0.980
The obvious causal relationship with the administration of the drug	33	29	62	20.3%	0.698
Discouraging Factors					
The low degree of severity of a clinical reaction	99	65	164	53.8%	0.060
Uncertainty regarding the type of reactions to be reported	81	53	134	44.0%	0.117
The uncertainty of a causal relationship with the administration of the drug	52	47	99	32.5%	0.482
A lack of information from the affected patient	57	42	99	32.5%	0.598
The reaction is widely known	48	30	78	25.6%	0.207
A lack of knowledge regarding the regulations and procedures for reporting	44	20	64	21.0%	0.016
The difficulty in obtaining a form for reporting	36	32	68	22.3%	0.642
The complexity of the form to be completed	33	22	55	18.0%	0.449
The fear of medical-legal consequences	30	17	47	15.4%	0.207
Reporting does not seem worthwhile	22	26	48	15.7%	0.146
A lack of time to report reactions due to heavy responsibilities	54	33	87	28.5%	0.139
A lack of support from your organization/head of department/colleagues	43	29	72	23.6%	0.400

**Table 6:** Participants’ perceptions of ADR types to report and aims of spontaneous suspected ADR monitoring and reporting (*N* = 305).

	HCP (*n* = 169)	CP (*n* = 136)	Total	% (of 305)	*p*-Value
Types of ADRs perceived to be reportable					
Suspected reactions	103	75	178	58.4%	0.307
Certain [sure, ascertained] reactions	98	69	167	54.8%	0.206
Severe reactions	88	67	155	50.8%	0.626
Mild reactions	55	34	89	29.2%	0.150
Reactions to drugs that have been in use for a long time	45	26	71	23.3%	0.123
Reactions to new drugs	62	41	103	33.8%	0.230
Known reactions	28	20	48	15.7%	0.657
Unexpected/unusual reactions	67	44	111	36.4%	0.188
Interactions between drugs	42	32	74	24.3%	0.789
Teratogenicity phenomena	54	34	88	28.9%	0.183
Reactions to vaccination	64	36	100	32.8%	0.035
Lack of efficacy due to resistant strain	61	31	92	30.2%	0.012
Aims of monitoring the spontaneous reporting of suspected ADRs					
To measure the incidence of ADRs	129	86	215	70.5%	0.013
To identify the indication for which the drugs are prescribed	102	76	178	58.4%	0.431
To identify factors predisposing patients to ADRs	93	65	158	51.8%	0.209
To identify uncommon ADRs (allergic, idiosyncratic, etc.)	74	57	131	43.0%	0.742
To identify previously unknown ADRs	40	41	81	26.6%	0.203
To identify safe drugs	65	49	114	37.4%	0.663
To maintain a database of ADRs	76	52	128	42.0%	0.236

## Data Availability

The original contributions presented in this study are included in the manuscript file. Further inquiries can be directed to the corresponding author.
